# Surgical Repair of Proximal Hamstring Tendon Avulsion

**DOI:** 10.1055/s-0045-1810038

**Published:** 2025-09-08

**Authors:** Guilherme Guadagnini Falotico, Bruno Francesco Scatigna

**Affiliations:** 1 Hip Group, Department of Orthopedics and Traumatology, Escola Paulista de Medicina, Universidade Federal de São Paulo, São Paulo, SP, Brazil

**Keywords:** hamstring muscle, hamstring tendons, tendon injury, lesões dos tendões, músculos isquiossurais, tendões dos músculos isquiotibiais

## Abstract

Injuries to the proximal hamstring muscle complex are common in athletes and range from strains to tendinous and bony avulsions. The lesion mechanism typically involves an eccentric contraction of the hamstring muscles during abrupt hip hyperflexion with the knee in extension. Low-speed injuries occur in high kicks and splits, whereas tendon avulsions are common in high-speed activities, such as running and ballet. Clinically, patients present with pain, subcutaneous hematoma, and, sometimes, a palpable defect. Additional signs include limited knee extension and involvement of the sciatic nerve. Diagnosis relies on ultrasonography, magnetic resonance imaging (MRI), and radiography, and MRI is the standard test. Surgical treatment is indicated for complete avulsions, especially in athletes, to prevent loss of strength and difficulty in returning to sports. In the surgical technique herein described, we perform one or two transverse incisions in the gluteal fold, depending on the tendon retraction, followed by fixation with metal anchors. The postoperative period includes initial restriction, followed by accelerated rehabilitation for return to sports by the twelfth week. Since 2019, the technique has been applied to 13 patients, demonstrating good outcomes, without re-ruptures and a postoperative Tegner score similar to the preoperative one.

## Resumo

As lesões do complexo dos músculos isquiotibiais proximais são frequentes em atletas e variam de distensões a avulsões tendíneas e ósseas. O mecanismo de lesão geralmente envolve contração excêntrica dos isquiotibiais durante hiperflexão abrupta do quadril com o joelho estendido. Lesões de baixa velocidade ocorrem em chutes altos e espacates, ao passo que avulsões tendíneas são comuns em atividades de alta velocidade, como corrida e balé. Clinicamente, os pacientes apresentam dor, hematoma subcutâneo e, em alguns casos, defeito palpável. Sinais adicionais incluem limitação na extensão do joelho e comprometimento do nervo ciático. O diagnóstico é feito por ultrassonografia, ressonância magnética (RM) e radiografia, sendo a RM o exame padrão. O tratamento cirúrgico é indicado para avulsões completas, especialmente em atletas, e visa evitar perda de força e dificuldades no retorno ao esporte. A técnica cirúrgica aqui descrita utiliza uma ou duas incisões transversais na prega glútea, dependendo da retração do tendão, com fixação por âncoras metálicas. O pós-operatório inclui restrição inicial, seguida de reabilitação acelerada para retorno ao esporte a partir da décima segunda semana. Desde 2019, a técnica foi aplicada em 13 pacientes, e demonstrou bons resultados sem rerrupturas, com pontuação pós-operatória no escore de Tegner semelhante à do pré-operatório.

## Introduction

Injuries to the proximal hamstring muscle complex pose a challenge to physically-active individuals and competitive athletes. The severity of these injuries can range from strains to complete myotendinous ruptures, proximal hamstring tendon avulsions, and bone avulsions. 1 2 The lesion mechanism usually involves eccentric contraction of the hamstrings secondary to abrupt hip hyperflexion with the knee in extension. 3 4 Proximal hamstring myotendinous rupture typically occurs in low-speed injuries, such as high kicks, splits, and sliding tackles. 3 In contrast, proximal hamstring tendon avulsions are frequent in high-speed activities, such as running, water skiing, or extreme range of motion in ballet. 4

Clinically, the patients present with pain, subcutaneous hematoma, and, sometimes, a palpable defect along the hamstring tract. 5 Additional signs include pain in knee extension in a sitting position, lack of hamstring muscle tension (bowstring sign), and contiguity-related involvement of the sciatic nerve, which may lead to motor and/or sensory deficits and neuropathic pain. 6 Diagnostic confirmation relies on several imaging modalities, including ultrasound, magnetic resonance imaging (MRI), and conventional radiograph, to assess bone involvement. The MRI is the most frequent method for diagnosis. 7

Complete tendon and bone avulsions represent a potential indication for surgical treatment, especially in competitive athletes, to avoid long recovery periods that could compromise their careers. Non-surgical treatment results in lower satisfaction rates, reduced hamstring muscle strength, and a lower likelihood of returning to preinjury sports levels. Postoperative care should prioritize initial protection of the repair, followed by an accelerated rehabilitation protocol for early return to sports at least 12 weeks after surgery. 8 9

The present study aimed to describe an open-repair technique for complete tendon avulsions through a transverse incision in the gluteal fold for injuries with up to 5 cm of retraction and 2 transverse incisions for cases with tendon stump distal migration greater than 5 cm. The discussion on surgical treatment remains scarce in the Brazilian literature, and there is no technique commonly performed by Brazilian orthopedists.

## Technical Description

The technique herein described is based on the experience with a series of 13 consecutive patients operated on by the same surgeons together (GGF and BFS). Table 1 shows epidemiological data, injury classification, and injury and follow-up times.

**Table 1:** Epidemiological data, lesion classification and time, and follow-up time

Sex	Age (years)	Time since lesion	Sport	Classification	Preoperative Tegner score	Postoperative Tegner score	Follow-up time	Complications
Female	27	11 weeks	Running	3	7	7	42 months	None
Male	22	3 weeks	Rugby	2C	10	10	33 months	None
Male	46	7 weeks	Bodybuilding	3	4	4	46 months	None
Male	48	2 weeks	Running	3	5	6	6 months	None
Male	53	2 days	Water skiing	3	6	6	22 months	None
Female	16	4 weeks	Judo	2C	9	7	14 months	Granuloma in the suture thread, with surgical reapproach
Female	43	6 weeks	Bodybuilding	2C	5	Not available	1 month	No acute complications
Male	52	2 weeks	Soccer	2C	5	Not available	1 month	No acute complications
Female	49	10 days	Cycling and bodybuilding	3	6	6	60 months	None
Male	34	12 weeks	*Capoeira*	1B	9	9	48 months	Pain recurrence with no signs of new lesion; the patient continued practicing sports
Male	14	10 days	Basketball	3 (avulsion fracture)	7	7	24 months	Loosening with migration of synthesis material requiring revision – no intercurrences after the revision
Male	30	8 weeks	Mixed martialarts (MMA)	3	9	9	4 months	None
Male	47	6 weeks	Olympic and artistic gymnastics	2C	9	Not available	1 month	None

The mean age of the patients was of 37(±13.2) years, ranging from 14 to 53 years. Regarding sex, there were 9 (69.2%) male patients. In total, 7 patients (53.8%) presented complete tendon avulsion and retractions greater than 2 cm (type-3 injury).

We used the Forlizzi et al. 10 (2022) classification to describe injuries as types 1A, 1B, 2C, 2S, and 3 (Appendix 1). The Tegner score (Appendix 2), a widespread functional assessment scale in orthopedics and physiotherapy, was used to measure physical and sports activity levels to assess sports performance. Tegner and Lysholm 11 developed this tool in 1985 as a complement to the Lysholm score to evaluate the ability to return to sports and work activities after knee injuries, but it is currently applied to other joints as well. The score ranges from 0 to 10, with 0 indicating inability to work or play sports resulting from a joint condition, and 10, participation in high-impact competitive sports (including professional soccer and rugby).

### Technique



**Video 1** Test after metallic anchor insertion.


**Video 2** Tendon suture.


**Video 3** Jumping activity during rehabilitation.


**Video 4** Strengthening of the hip muscles during rehabilitation.


The patient remains in the prone position with protective pads on the chest and knee flexion at approximately 45° (to bring the stump closer and relax the sciatic nerve) under spinal anesthesia and sedation. The incision follows the gluteal fold and extends for 5 to 7 cm, depending on the patient's muscle volume. After skin and subcutaneous tissue opening, we identify and dissect the gluteus maximus fascia to avoid injury to the posterior femoral cutaneous nerve. After opening the fascia and protecting the nerve, we identify the tendon stump and handle it with care due to its proximity to the sciatic nerve. After identifying the tendon, we perform digital neurolysis of the sciatic nerve and isolate the stump for later repair. Ischial tuberosity scarification provides better tendon healing. We routinely use three 5.5-mm metal anchors to mimic the original footprint of the tendon (1 anchor for the semimembranosus tendon and 2 for the conjoint tendon) in continuous sutures and anchoring per the Krackow method.

In cases with significant retraction (greater than 5 cm after patient positioning) and injury time longer than 3 weeks, we can add a second transverse incision over the tendon stump projection to release the fibrosis and facilitate tendon sliding to the proximal region, minimizing the risk of sciatic nerve injury.

The incision is closed in layers, as is usual in other procedures. The postoperative period should respect suture protection, using a partial weight-bearing protocol with crutches for 2 weeks and restricting combined hip flexion and knee extension movements. It is possible to accelerate rehabilitation after the fourth week and allow the return to sports by the twelfth week.

Figures 1 to 9 and Videos 1 2 3 to 4 illustrate the surgical steps.

**Fig. 1:**
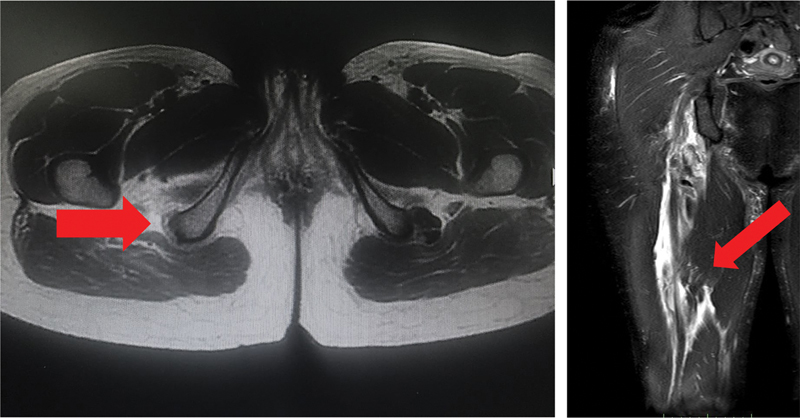
Axial section showing the ischial tuberosity without the presence of the conjoint and semimembranosus tendons; coronal section showing a large amount of fluid and retraction of the tendon stump.

**Fig. 2:**
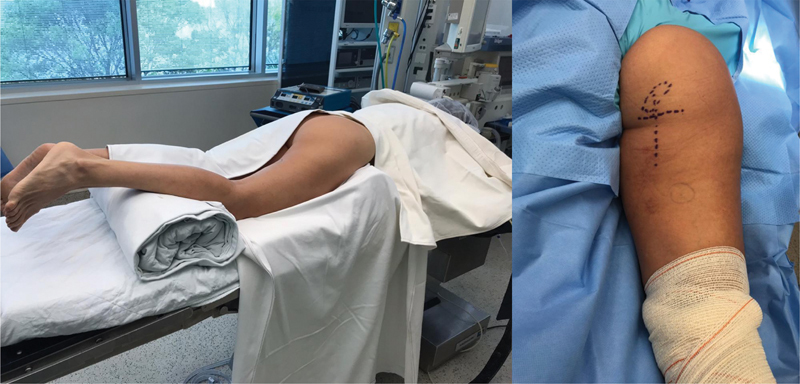
Positioning for surgery – patient in prone position with cushion under the knees to reduce tension on the tendon stump and sciatic nerve; planning of the transverse incision in the gluteal fold and longitudinal projection of the stump retraction.

**Fig. 3:**
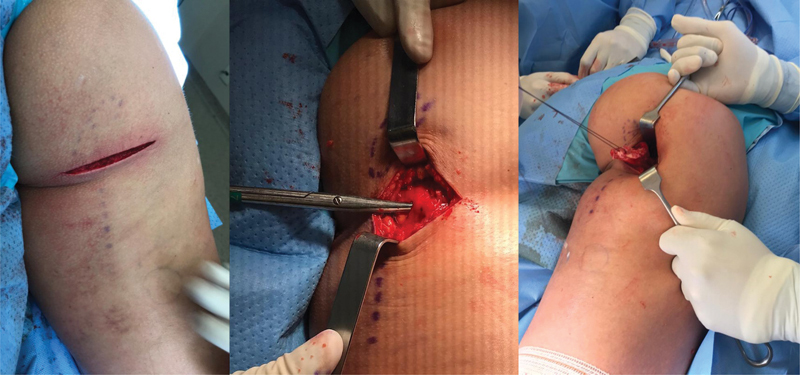
Skin and subcutaneous incision; dissection of the posterior femoral cutaneous nerve near the gluteus maximus fascia; identification and repair of the tendon stump.

**Fig. 4:**
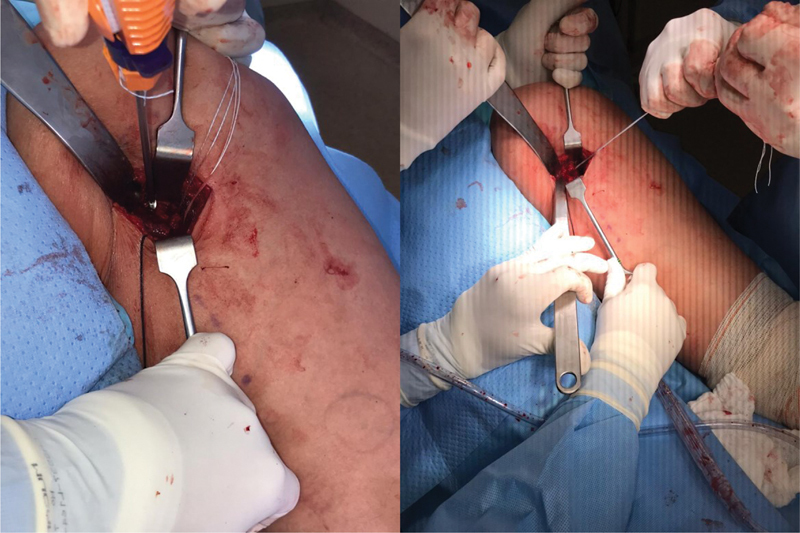
Insertion of 5.5 mm metal anchors into the ischial tuberosity; 3 anchors used in the footprint (2 for the conjoint tendon and 1 for the semimembranosus)

**Fig. 5:**
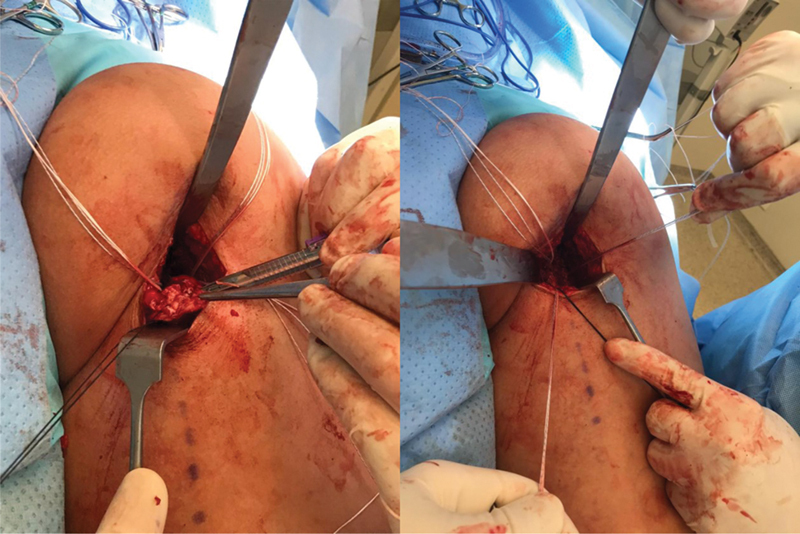
Suturing the tendon with continuous anchored stitches and reinforcement with simple stitches.

**Fig. 6:**
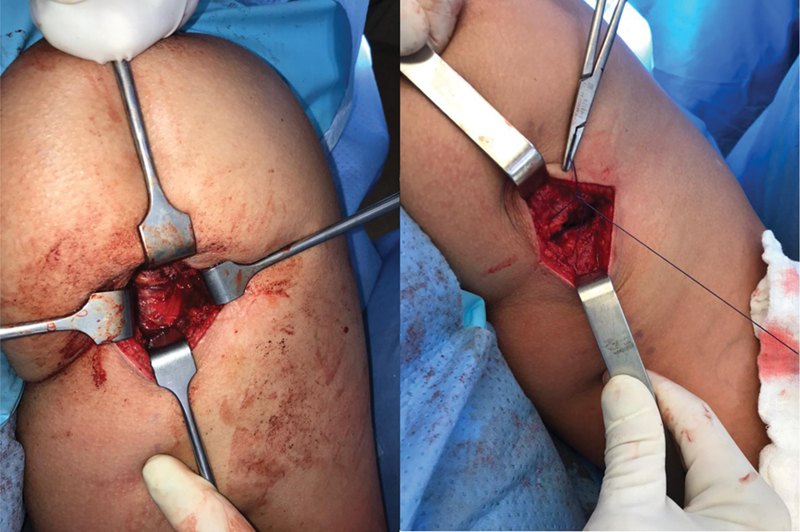
Tendon stump reinserted into the footprint; closure of the fascia to prevent adhesions.

**Fig. 7:**
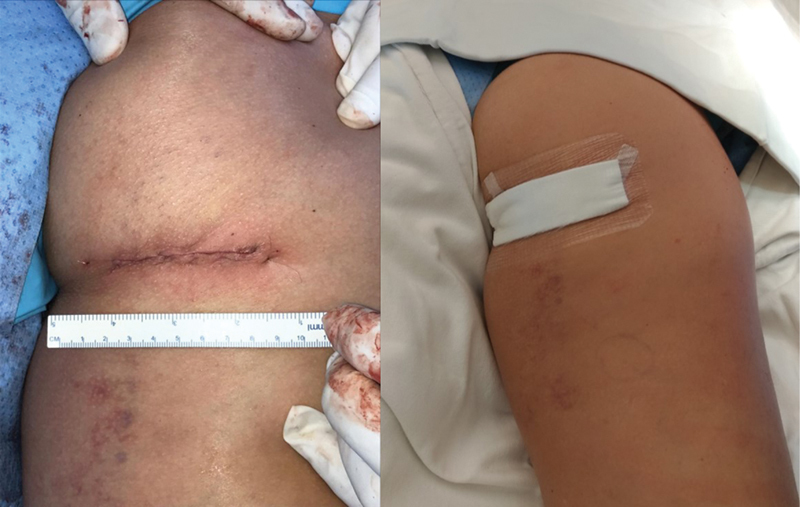
Cosmetic appearance of the suture; waterproof dressing used to prevent secondary contamination.

**Fig. 8:**
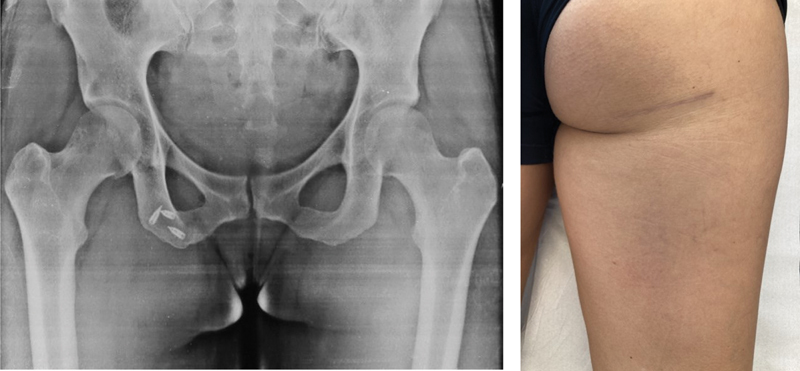
Final radiographic appearance with anchors in the tendon footprint; scar 3 months postoperatively.

**Fig. 9:**
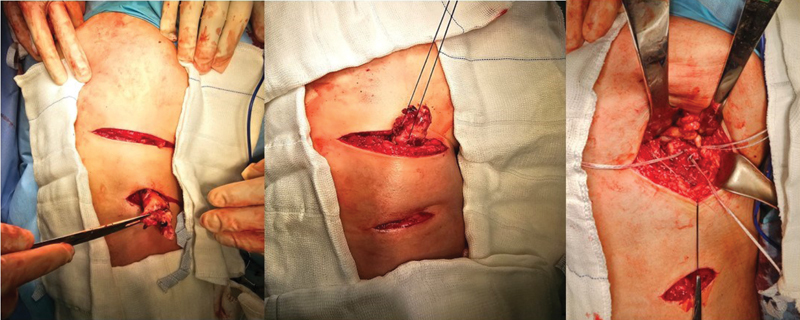
Lesion at 12 weeks and 11 centimeters of distal retraction; double transverse incision technique performed.

## Final Comments

We have been using this technique since 2019, performing surgical repair in 13 patients during this period. The surgery has proven to be reproducible, resulting in good clinical outcomes, with no cases of re-rupture to date and a postoperative Tegner score similar to the preoperative one. As the Shapiro-Wilk test showed that the data did not present normal distribution, we used the nonparametric Wilcoxon test for paired samples. This test revealed a mean preoperative Tegner score of 7.1 ± 1.97 and a mean postoperative Tegner score of 7.0 ± 1.83 ( *p* = 0.72), demonstrating good sports recovery capacity.

The usual surgical technique for patients with retractions greater than 5.0 cm is a longitudinal incision, a wide approach with a higher potential risk of suture dehiscence. The transverse technique presented here, with one or two incisions, is feasible and an alternative for the surgical treatment of proximal hamstring injuries.

The main surgical indications included complete tendon avulsion or conjoint tendon avulsion with retraction greater than 2 cm in physically-active patients under 65 years old.
